# A New Condition Associated with Hemorrhages in the Bladder Wall and Urinary Symptoms

**Published:** 1919

**Authors:** 


### UROLOGY AND DERMATOLOGY

### *A New Condition Associated with Hemorrhages in the Bladder Wall and Urinary Symptoms. Preliminary Report.* By JOHN T. PETERS, Captain M. R. C., and A. RAYMOND STEVENS, Captain M. R. C. *Journal of the American Medical Association.*

Of the 155 patients examined, 73 were found to be suffering from chronic cardiac or renal disease, with a recurrence of symptoms; 58 gave pretty clear evidence of previous cardiac or renal disease; 7 gave no history of previous cardiac or renal symptoms, but suggested in their course an underlying chronic affection. Of the remaining 82 cases, 49 presented a syndrome marking them definitely as an acute nephritis. A small group of 8 patients developed signs of nephritis in the course of what appeared to be typical trench fever, but whether or not the trench fever bore any causable relation to the nephritis, it is impossible to say. Four cases of panurinary infection were found. A patient was received with profuse hematuria who complained of symptoms strongly suggestive of vesical calculus or papilloma. Cystoscopic examination by one of the authors revealed neither stone nor neoplasm, but instead multiple submucous hemorrhages in the bladder wall, and blood pouring from both ureters. These patients presented predominant symptoms, and the only constant ones were profuse hematuria, frequency of urination, and pyrexia of an irregular and variable type. The majority also had pain on urination. In most cases the initial temperature was quite high, the curve was very irregular, and no definite type appeared.

With the possible exception of one patient, who had had general edema, the cardiac and renal history may be considered negative.

